# U.S. Antitrust Policy in the Age of Amazon, Google, Microsoft, Apple, Netflix and Facebook

**DOI:** 10.1007/s10602-022-09391-9

**Published:** 2023-03-20

**Authors:** Thomas W. Hazlett

**Affiliations:** 1 John E. Walker Department of Economics, Clemson University, Clemson, SC USA; 2 Hoover Institution, Stanford University, Stanford, CA USA

**Keywords:** Antitrust policy, Digital platforms, Vertical integration, L40, L50, L96, K21

## Abstract

Sweeping changes have disrupted society courtesy of the Information Revolution, presenting great opportunities in radically transformed economic markets but also great challenges in adapting to new and different forms of organization. Antitrust laws and other elements of competition policy are being re-examined. Specifically, the House Judiciary Committee conducted hearings in 2020 in which it asked key questions about the pattern of development in U.S. markets and options for policy reform. This paper, answering such queries, finds strong evidence for the view that, relative to practical alternatives that include E.U.-style regulation, digital markets in the U.S. appear robust, generating considerable innovation that produces pro-consumer outcomes. The global Internet is dominated by U.S.-developed technologies and business models discovered and deployed in a process of competitive rivalry. Even given imperfect rules and regulations, U.S. markets have contributed strongly to economic advances embraced around the world.

## Introduction

Are U.S. digital markets advancing, or threatening, the American economy? There is keen interest in the answer to this question. Sweeping changes have disrupted society courtesy of the Information Revolution, presenting great opportunities in radically transformed economic markets but also great challenges in adapting to new and different forms of organization. Great hope accompanies the former, much concern attends the latter. Now important discussions are engaging as to the impacts of market power, where competitive forces—beneficial in discovering new efficiencies and promoting Consumer Welfare—may be thwarted. Antitrust laws and other elements of competition policy are being re-examined.

Specifically, the House Judiciary Committee has asked for comment on the following:

1. The adequacy of existing laws that prohibit monopolization and monopolistic conduct, including whether current statutes and case law are suitable to address any potentially anti-competitive conduct;
2. The adequacy of existing laws that prohibit anti-competitive transactions, including whether current statutes and case law are sufficient to address potentially anti-competitive vertical and conglomerate mergers, serial acquisitions, data acquisitions, or acquisitions of potential competitors; and
3. Whether the institutional structure of antitrust enforcement—including the current levels of appropriations to the antitrust agencies, existing antitrust authorities, congressional oversight of enforcement, and current statutes and case law—is adequate to promote the robust enforcement of the antitrust laws.

This paper attempts to inform the answers to these questions by examining industrial concentration in the information economy and its impact on U.S. market competition; vertical integration in digital platforms; forces driving innovation in business models and product markets; and the use of competition policy tools in high tech markets. These latter include broadband access and mobile phone industries, which have attracted attention as specific examples of areas where the U.S. has is seen by some as lagging rival economies due to insufficient antitrust enforcement.

The evidence supports the view that, relative to practical alternatives that include E.U.-style regulation, digital markets in the U.S. appear robust, generating considerable innovation that produces pro-consumer outcomes. The global Internet is dominated by U.S.-developed technologies and business models, discovered and deployed in a process of competitive rivalry. The emergent markets in online services and e-commerce have created enormous efficiencies and valuable new services,^1^ rewarding consumers^2^ and reconfiguring numerous industries, as users adopt preferred ways of working, shopping, learning, and enjoying entertainment media. This has not ended our social problems; every innovation introduces complications as we adjust to change.^3^ We will rightfully be aware of the challenges and be alert to policy reforms that improve welfare. These are appropriately addressed in a framework that continues to facilitate new options. Communications networks and digital services have massively increased information flows and the opportunities for gains from trade. Even given imperfect rules and regulations, U.S. markets have contributed strongly to economic advances embraced around the world.

Antitrust policies govern and have been applied. *U.S. v. AT&T* led to the divestiture of the world’s largest corporation in 1984, helping markets overcome anticompetitive barriers (including those established by FCC regulation) in the years to follow.^4^ On the other hand, the AOL-Time Warner merger, the largest in history (when proposed in 2000 and now), was opposed by the Federal Trade Commission. The remedies the FTC applied to clear the merger proved irrelevant to post-merger markets, as did the market power argument asserted to thwart the combination.^5^ More recently, the Department of Justice sued to block the AT&T/T-Mobile wireless network combination in 2011, succeeding in its action (the merger was abandoned); regulators later commented positively on this effort and its outcome.^6^

In 2017, the DOJ sued to prevent AT&T’s acquisition of Time Warner. Its argument was rejected by federal courts because the evidence did not support the allegation that the merger would harm consumers. The careful analyses performed in the District Court verdict^7^ and then in the ruling of the D.C. Court of Appeals^8^ make good readings to assign in economics courses.^9^ They suggest that, if the Government sometimes fails to bring all the antitrust cases it might, it also errs by in bringing cases it should not.

U.S. policies have managed to incentivize great progress in high tech markets.

Distributed IP networks were privatized in the early 1990s, and left to develop via market forces,^10^ a distinct approach to that envisioned by, for instance, France Telecom’s Minitel. Mass market access to the Internet became popular first in the U.S., rapidly spreading by competitive forces allowed to flourish, as common carrier obligations for entrants such as AOL were abandoned.^11^ Residential broadband markets then emerged in rivalry between (unregulated) cable TV operators and telecommunications carriers. Bringing high-speed data services to the home has opened whole new sectors of the New Economy. Wireless mobile networks, first launched in the U.S., have rapidly replacing landline phones in affluent countries, and long ago did so in developing markets, where the vast majority of the population never gained access via the traditional PTTs—state owned monopolies controlling postal, telegraph and telephone services (infamous for their years-long phone install waiting lists). U.S. mobile markets have proven competitive from an international perspective. Americans consume more mobile data per capita do residents in any large European country. There are many public policies that would yet enhance American competitiveness.^12^ Rejecting open markets in favor of more highly regulated systems, or pushing antitrust law away from its current focus on Consumer Welfare,^13^ are not likely to be among them.^14^

## Evolving antitrust rules

Antitrust rules were established under common law, even prior to enactment of the Sherman Antitrust Act in 1890. But with the rise of Big Business, concern over large industrial enterprises took new legal form. In our time, another economic transformation is credited with sparking a similar policy debate. With the rapid evolution of digital technologies, we are witness to the dramatic rise of communications, social media, and e-commerce platforms, extremely popular with users and prized by financial investors. They have enabled far-reaching business model innovation and disrupted many markets. They present new opportunities and new challenges. As with the printing press, the telegraph, photography, motion pictures, and radio and television, rules governing these new institutions are evolving in response to changing circumstances.

Each generation seems to face the challenge. In historical terms, Walmart was only recently the object of policy makers’ interest. A store that computerized supply lines and slashed costs with “just in time” inventories, engineering new economies to extend low prices to communities disadvantaged under traditional terms of term, sparked controversy. Walmart was seen as a giant, integrated firm that was unfairly competing with smaller, local enterprises. In some sense that was true: the firm was a vehicle of “creative destruction,” the method in which capitalist economies lurch forward to higher levels of productivity.

But the net gains far outweighed alternatives, as Walmart fashioned a middle-class discount experience that New York University (and, later, Obama Administration) economist Jason Furman called “a progressive success story.”^15^ That was on economic research that Walmart’s value proposition, as of 2005, awarded over $250 billion annually to relatively low-income consumers, dwarfing the Food Stamp program ($33 billion) or the Earned Income Tax Credit ($40 billion). As Sebastian Mallaby wrote of the campaign to stop additional Walmart stores from being built:

Only by summoning up the most naive view of corporate behavior can the critics be shocked - shocked! - by the giant retailer's machinations…. Wal-Mart aims to enrich shareholders and put rivals out of business! Hello? What business doesn't do that?

If critics prevent the firm from opening new branches, they will prevent ordinary families from sharing in those gains. Poor Americans will be chief among the casualties.^16^

Walmart offered solutions embraced by millions: “trade-tested betterment,” in Deirdre McCloskey’s nimble phrase.^17^ Sears, Montgomery Ward, A&P, Safeway and the low-price, national chains created similar advances via economies of scale generations before—and triggered the same hostilities. Indeed, they inspired antitrust legislation in the 1936 Robinson Patman Act which—as described by another product of global economies of scale, Wikipedia—“was designed to protect small retail shops against competition from chain stores by fixing a minimum price for retail products.”^18^

The Sherman Act of 1890 did not “outlaw monopolies.” Where outsized firms arise due to superior performance they are pro-consumer and antitrust law has—particularly when done correctly—accommodated. Supreme Court Justice William O. Douglas wrote in 1948 that "large scale buying is not, of course, unlawful per se*.* It may yield price or other lawful advantages to the buyer.”^19^ This is the short answer to why the Department of Justice has not sued to break-up Amazon, Facebook, Google or Apple. The reality is that improving upon market outcomes via aggressive antitrust actions is difficult.

The “error-cost framework” has come to dominate legal thinking due to the reality that mistakes can occur from too much antitrust as well as too little.^20^ “False positives” incorrectly prohibit efficient, or at least benign, market actions. These errors may “have resounding chilling effects,” write Joshua Wright, Elyse Dorsey, Jonathan Klick, and Jan Rybcinek (2019, 305–306) deterring “the condemned firm from engaging in … beneficial conduct.”^21^

MIT's Industrial Productivity Commission, headed by Nobel economist Robert Solow, reported in 1989 that U.S. innovation, particularly that involving large-scale platforms, "has often, though not always, been inhibited by government antitrust regulation."^22^ UC Berkeley scholars Tom Jorde and David Teece add:

If innovating firms do not have the necessary capabilities in-house, they may need to engage in various forms of restrictive contracts with providers of inputs and complementary assets. The possibility that antitrust laws could be invoked, particularly by excluded competitors, thus arises. Lying in the weeds to create mischief for unsuspecting firms engaged in socially desirable but poorly understood business practices are plaintiffs' attorneys and their expert economists entreating the courts to view reality through the lens of monopoly theory and modern variants such as raising rivals [costs].^23^

Some may believe that there was a Golden Era in Antitrust that we might recapture. That is a chimera. In a survey of the last century’s largest antitrust cases published in the *Journal of Economic Perspectives*, Brookings Institution regulatory economists Robert Crandall and Cliff Winston describe the pattern: cost-effective remedies remain elusive. The policy options include structural remedies, such as divestitures; behavioral rule; commission-type “public interest” regulation; or other court-imposed standards. Even today, 130 years after the Sherman Act created federal rules (and Department of Justice enforcement) for competition policy, and 106 years after the Federal Trade Commission was born, to assist in the task, policy makers still debate, for instance, whether behavioral rules should be used at all.^24^

Crandall and Winston focus on six mega monopolization cases, listed in Table 1.^25^ They show that *Standard Oil*, breaking up John D. Rockefeller’s trust, attacked concentration in petroleum refining only when “Standard's alleged market power had already declined substantially … from 82 percent in 1899 to 64 percent in 1911.”^26^ More ominously, “Retail prices rose with divesture,” as shown statistically. The 1911 divestiture of cigarette makers following *American Tobacco* fared better—no measurable impact on retail prices. Ditto for the 1945 *Alcoa* case (Table 2).

**Table 1:** Outcomes of Biggest, Successful U.S. Antitrust Cases (Crandall & Winston, 2003)

Case	Date	Violation	Remedy	Effect
Standard Oil	1911	Predation, monopolization of refined oil	Divestiture split into 38 firms	“little effect”; retail prices rose by a statistically insignificant amount; equity share prices unaffected
American Tobacco	1911	Predation, monopolization of tobacco	Divestiture split into 3 firms	“did little to spur meaningful competition”; advertising up, but no effect on retail prices, farm (wholesale) prices, or industry profits
Alcoa	1945	Monopolization of aluminum	Divestiture split into 3 firms	“no effect on real aluminum prices”
Paramount	1948	Vertical foreclosure of independent theaters	Divestiture split movie distributors from theaters	“average real price of a movie ticket rose [for] two decades”
United Shoe Machinery	1954	Monopolization by tying	USM forced to sell, not rent, machines	Lower concentration but continuation of United’s dominance and profits; 15 years later Supreme Court imposed divestiture
AT&T	1982	Monopolization by tying	AT&T split into 8 major parts	Positive competitive impacts, but unrelated to the divestiture; largest impact was in reducing FCC regulatory barriers

The *United Shoe* case, decided in 1949, has been assessed by U.S. courts themselves: “the U.S. Supreme Court was not satisfied that sufficient competition had developed in the shoe machinery market, because following a review of the decree, it recommended in 1969 that the lower court consider "more definitive means" to achieve competition.”^27^

Perhaps the most apt illustration of antitrust policy in action occurs with *U.S. v. AT&T*. The phone monopolist was a regulated common carrier, and U.S. rules under the Federal Communications Commission had pre-empted competitive entry. Despite an ostensible “open platform,” mandating non-discriminatory access by customers and competitors (to AT&T), the FCC had imposed restrictions that protected Ma Bell’s market and flummoxed upstart rivals. The case did succeed, in some measure, in moving the country to a more competitive environment. But it is the exception^28^ that proves the rule. As Crandall & Winston write: “Thus, antitrust policy did not triumph in this case over restrictive practices by a monopolist to block competition, but instead it overcame anticompetitive policies by a federal regulatory agency.”^29^

## Horizontal concentration

### Efficiency and scale

The Digital Economy sees disruptive events with some frequency, and it appears a commonplace that entrants may destroy established giants.^30^ Many of these innovations leverage efficiencies available from the creation of large-scale platforms. This is not a new phenomenon, but dates at least to the Industrial Revolution. With advances in communications and transportation networks, as well as the deployment of increasingly advanced agricultural and factory technologies, distinct economies of scale were discovered. These allowed increases in labor productivity and raised living standards spectacularly, leading to “the Great Enrichment,”^31^ the history-bending ascent to affluence which society now enjoys.

Efficiencies from scale increases are not automatic, but they can be highly beneficial. Proposing to categorically restrict them—as did the late Supreme Court Justice Louis Brandeis, who opposed low prices and even volume discounts—is a dangerously anti-consumer policy.^32^ Thomas McCraw’s Pulitzer Prize winning history of regulation provides a profound explanation of Brandeis’ antitrust philosophy, finding that his “deep-seated antipathy toward bigness clouded his judgement.” And it led to economic error. “Instead of drawing the correct conclusion—that large size was an advantage to firms in some types of industries and a disadvantage to firms in other types—Brandeis too simply asserted that bigness in general was inefficient.”^33^

Nobel Laureate Oliver Williamson contributed a crucial counter argument to Brandeis’ categorical view in 1968, showing that scale economies should be explicitly considered in merger cases. Combinations where enhanced size would unleash productive gains would increase social welfare and yield dynamic gains by giving firms stronger incentives to create more efficient structures. This view became widely accepted by lawyers, judges and economists as sound policy.^34^ This movement was furthered by empirical analysis showing that markets with high concentration generally appeared to become concentrated due to the expansion of relatively efficient firms. This suggested that the positive correlation between concentration and profitability, as in the “Structure-Conduct-Performance” paradigm in industrial organization, was due to the deployment of efficiencies rather than monopolistic restrictions. The full story was Schumpeterian: the quest for profitable innovation was driving commercial success and simultaneously enhancing pro-consumer efficiencies.^35^

### Dynamic innovation in business models and platforms

Ben Thompson, author of Stratechery,^36^ offers insights into how high-tech firms pursue various strategies, jockeying for market position and profits. An important meme in tech investment, explains Thompson, is the “franchise,” the business idea that

(a) successfully meets a need;

(b) costs less to provide than what consumers will pay;

(c) is not easily duplicated by rivals.

This is the simple logic of value creation. In an entrepreneurial process, improvements are made in existing opportunities, and they are measured as successful if these changes generate returns in excess of opportunity costs. Better investments are those ideas that create and then sustain these achievements. Incentives of entrepreneurs, investors and society are well aligned.

In the quest to achieve the last of these conditions, sustaining profitability in the face of possible entry by profit-seeking rivals, entrepreneurs seek to create a unique asset that can exert competitive superiority over rivals, perhaps protected by intellectual property rights and/or a “first mover” advantage. These claims are then evaluated by potential investors, with market trading revealing an equilibrium among differing opinions and establishing the market value of the firm. Ben Thompson focuses on the source of sustainability for tech firms, comparing Uber to Amazon, where a “big problem” arises.

with contractor-reliant businesses like Uber… is how much time and money they need to spend on the supply side of the equation to acquire and retain drivers. To be fair, this is in large part due to competition: when both riders and drivers can switch freely the former need lower prices and the latter higher incentives, both of which comes at the cost of Uber’s finances. Indeed, this may simply be an old-fashioned missing moat problem.^37^

The “moat” is the franchise.^38^ While market power critics tend to categorically identify the moat as a barrier to entry that restricts competition, and in some cases it may well be, in a dynamic process of innovation the investor’s view is Schumpeterian (and Thompson’s point): the competition for investments is seeking out the best *value creating* activities. To see the profitable outcome of an innovation process as monopolistic, without counting the “creative” side of the “destruction,” is to enter this story in the middle, a point made compellingly by Harold Demsetz (discussed below).

There are, of course, countless ways to potentially produce the efficiencies that such market niches aim to realize. E-commerce platforms have been successfully launched by e-Bay and Amazon, with characteristic similarities and yet deep distinctions. Most obviously, e-Bay is less vertically integrated, offering an online store where retail sales are made by other parties, whereas Amazon owns not only the wholesale platform but actively participates in retailing, competing with its seller-customers.

When disruptive technologies do perform a valuable service, as determined by the competitive market test, they can generate enormously popular and useful gains at surprisingly low cost (in capital investment and operational expense). Thompson alerts investors to look for high leverage opportunities, again consistent with society’s desire to prosper, discovering low outlays might result in the highest returns. In tech market terms, Thompson notes.

the smaller a piece that software plays in a company’s overall offering, the more difficult it is to introduce network effects or ecosystem lock-ins that reduce competition. You’re just selling another widget—as a service, of course. This makes Uber-type services very different than a marketplace like Ebay, which had huge network effects.

The leverage then comes from *economies of scale* that make a given infrastructure—supplied by software, a sales platform, reputational capital (as a “low price” seller or “America’s marketplace”) or all of the above—more advantageous for consumers. Thompson warns investors, reprising his Uber example, that the “smaller a piece that fixed cost investments (as opposed to operating expenses like rider and driver incentives) play in a company’s overall expenditure, the more difficult it is to build a more traditional distribution moat like Amazon. The result is that too many of these businesses are asset-lite like Ebay but without the network effects, even as they are heavy spenders like Amazon but without any actual assets to show for it.”

The competitive rivalry of firms in digital markets obeys the basic dynamics of firms in previous eras, but the possibilities for large scale economies tend to be richer. Digital platform audiences can be extremely large, given new efficiencies, and e-activities produced at great savings per unit. This is a replay of the Industrial Revolution, when emerging communications and transportation networks, combined with progress in science, allowed production from one firm to achieve lower costs by supplying far wider markets. The tension between local businesses and national chains intensified and became political. The basic source of this tension is not inefficiency, or restrictions in output tied to market power, but it’s reverse: lower prices via investments creating larger scale efficiencies than previously available.

### The problem of “starting in the middle”^39^

Harold Demsetz^40^ responded to the view of entry barriers that “tends to treat as unproductive the costs that must be incurred to create and to maintain a good reputation, to bear risks of innovation, and to build a scale of operations appropriate to the economical servicing of consumer demands, and it tends to neglect the incentives that will face future decision makers as a result of today's policy.” In failing to understand the efficiencies created, or the counter-factuals, spontaneous progress is not only under-appreciated but actively deterred—as with categorical limits on size or policies to impose divestitures.

Demsetz^41^ warns that “deconcentration may have the total effect of promoting inefficiency even though it also may reduce some monopoly-caused inefficiencies.” To impose policies bars setting market structures is dangerous work. And it misses the point. “[T]he maximization of competition is a meaningless goal. The goal is more accurately described as choosing a preferred mixture of competitive forms. Thus, price competition between existing goods can be intensified by eliminating patent and copyright protection, but this reduces the effectiveness of competition to produce new goods.”^42^

An appropriate economic inquiry examines competition for the market—perhaps winner-take-all, perhaps not, cycling through changing structures over time. That would bring into focus the differentiated strategies of many Amazon rivals (looping back to the discussion last section) including eBay, Walmart, Sears, Shopify, Etsy, Google, Yahoo!, America Online, Target, Circuit City, Best Buy, Instacart and others, some living and some dead. Barnes & Noble was an established incumbent in retail book sales when Amazon was created as an online book vendor in 1995. It threatened to “launch a website soon and crush Amazon” in 1996 when the upstart had $16 million in sales and the store-based bookseller some $2 billion.^43^ The B&N CEO wanted to call the new website “Book Predator” (a suggestion that was over-ruled), but it took months to construct. “[D]uring that time, Bezos’ team accelerated the pace of innovation and expansion.”^44^ Out of this contest emerged the triumphant upstart, Amazon.^45^

It was not because Amazon observed rules against pricing below average variable cost. It did, however, make investments in marketing and platform building—communicating a relentless pro-shopper policy like this:

As if to prove his singular obsession with customer experience, Bezos placed an expensive bet… In July [2000], author J.K. Rowling published the fourth book in the series, *Harry Potter and the Goblet of Fire*. Amazon offered a 40 percent discount on the book and express delivery so customers would get it on Saturday, July 8—the day the books was released—for the cost of regulator delivery. Amazon lost a few dollars on each of about 255,000 orders, just the kind of money-losing gambit that frustrated Wall Street. But Bezos refused to see it as anything other than a move to build customer loyalty.^46^

Amazon’s strategy of “get big fast” required just such outlays.^47^ The end of these efforts was to create widely shared facilities: (a) inventories leveraging volume discounts, (b) delivery networks supplying far quicker service than rivals; (c) easy-to-negotiate software interfaces, (d) superior customer service, (e) “big data” to assist customers in product search and to reduce shipping and handling costs.

Economists have long observed that some firms tend to grow large, and that the industries that host these large firms often exhibit relatively high profits. That led some to posit that the high concentration was causing the high profits, and that a deconcentration—through industrial policy, antitrust, regulation, or some other means—would increase output, lower prices, and improve social efficiency. This, in fact, was the view of the Chicago School in mid-Twentieth Century.^48^

Demsetz sought evidence on the causality assumed in the Structure–Conduct–Performance paradigm. The concentration-profits correlation had been shown to often be positive. The conclusion claimed by champions of the S–C–P model was that the higher concentration levels were driving the higher profit ratios, suggesting monopolistic output restrictions. Demsetz allowed as how that might be the case, but also that the concentration-profits correlation might be spurious. That is, the rise of more efficient firms, with lower costs and/or enhanced products, might logically exhibit higher growth, increasing industry concentration. In that case, the same concentration-profits correlation would be observed, but would best be explained by efficiency, not monopoly.

Examining profit levels across firms of different sizes in markets of given concentration provided a test. If high concentration were driving high profits, through collusion or output restriction, then firms of all sizes would tend to experience high profits. Yet if high concentration was related to economies of scale, then larger firms would exhibit higher returns than smaller rival firms.

This latter relationship is what the data tended to show. Demsetz concluded: “If rivals seek better ways to satisfy buyers or to produce a product, and if one or a few succeed in such endeavors, then the reward for their entrepreneurial efforts is likely to be some (short term) monopoly power and this may be associated with increased industrial concentration. To destroy such power when it arises may very well remove the incentive for progress.”^49^ The evidence was powerful, and the insight held up when tested by others.^50^ And recent research suggests that these trends are still observable in current changes taking place in the digital economy.^51^ By itself, the concentration-profits correlation began the story in the middle.^52^

The “reflexive antipathy towards even moderate concentration levels foundered in the 1970s,” write legal scholars Tim Muris and Jonathan Neuchterlein, “on the empirical evidence and, in particular, on the highly influential research of Harold Demsetz.”^53^ By the turn of the century, leading industrial organization texts noted that “the barrage of criticism [of the Structure-Conduct-Performance approach] has caused most research in this area to cease.”^54^

### Industry concentration ratios

Much of the current interest in antitrust policy is driven by studies that show certain measures of concentration are increasing in the U.S. economy. But concentration ratios, as given (and widely cited) in a May 2016 report from the President’s Council of Economic Advisers (“Benefits of Competition and Indicators of Market Power”), do not imply this. The analysis simply shows statistical changes that have no clear impact on competitive rivalry or consumer welfare.

In one instance, market shares for the Top Fifty firms in an industry are shown to be increasing over time in the U.S. But this does not inform how competition in the Digital Economy is progressing. How would the total elimination—say, through merger—of the 10^th^, 20^th^ or 30^th^ largest firm in a market impact the robustness of rivalry? If it did, it should be noted, defendants in antitrust suits would be delighted to hear of it: the top two or three firms in the industry could then claim that a merger they might arrange, for example, would be offset by the presence of so many small competitors.

In another measure of concentration in the CEA report some broad industries are shown to be decreasing in fragmentation. This asserted consolidation is of no competitive relevance given the business markets are not aligned with actual consumer choices. To see the problem, suppose that there are two cellular telephone operators licensed to serve each of 734 local markets, one having an “A” license, the other a “B,” and that all the companies are of equal size. (The markets do not overlap, and exhaustively cover the entire United States.) Everywhere you might live, you face a choice between two options: a duopoly. But when market concentration is calculated on a nationwide basis, the market would look highly competitive, with an HHI approximately equal to zero.^55^ Now suppose that each of the A licensees merge, forming a national A network. And, then, all the B licensees do the same. Two companies would remain, and supposing they were of equal size, the HHI would rise to 5,000 (more if they were not exactly equal). But consumers have the same number of choices: two. Indeed, the practical matter is that the mergers allowed national networks to emerge, and this would likely reduce certain costs, such as roaming charges or marketing expense, and increase the ease of mobility— a bonus in mobile services. From the standpoint of consumers, the competitiveness of both the A and B networks has not decreased and, reflecting real-world conditions, likely *increased.* This is roughly what happened after U.S. cellular licenses were distributed in each of 734 markets, two per area, in the 1980s. More to the point, it shows how changes in national HHIs may mischaracterize the competitive margins of interest.^56^

This is why economists have effectively critiqued such reported trends as somewhere between meaningless and misleading.^57^ Where increases in concentration, reasonably measured, are observed, there is no apparent harm. Take the measurements presented in a recent paper by the Brookings Institution.^58^ It displays increases in concentration estimated in six categories: Services, Manufacturing, Retailing, Wholesaling, Utilities and Finance, 1982–2012. By far, the largest increase is in Retailing, where concentration estimated to increase by 416%. The finding should indicate how uncompelling such broad measures of concentration are in flagging problem areas, because retail choice moved decidedly in favor of consumers in the decades studied.

Now, thanks to platforms offering “long tail” selections encompassing truly massive inventories, eCommerce platforms, and Google (or other) search engines to identify bargains, Americans have achieved something of a Golden Age in retail choice. Indeed, in considering the pros and cons of recent market developments from a competitiveness standpoint, Tyler Cowen says that the “good news” starts with retailing.^59^ He cites his ability to buy books on Amazon or eBay, or using online search to find other book sellers. “[M]y options as a book consumer have never been better.”^60^ Numerous efficiencies have also allowed far more discounters to compete: “Dollar General and Dollar Tree… had 27,465 outlets… more than the total number of CVS, Rite Aid and Walgreens stores combined.”^61^ In short, it is unclear how the steep increase in measured industrial concentration has raised prices for retail consumers. It is not particularly mysterious, however, that economies of scale—which could well be driving some concentration measures—has become more important in U.S. markets in recent decades. This is the cause and effect that is suggested by Brookings’ observation that “concentration is high in markets with large returns to scale and network effects.”^62^

It is also the observation of Eli Noam in his comprehensive 2009 study of “Media Ownership and Concentration in America.”^63^ He concludes that some digital markets show trends towards higher concentration, but there was widespread misinterpretation as to the origins and effects and of these changes. “[T]he structure of media are being transformed by broad forces, and concentration is its symptom, not its cause.”^64^ He observes that strong positions have been taken assuming causation the other way, even when no data support the position. He drills down on the late Ben Bagdikian’s popular and influential 2004 text, “Media Monopoly,” as presenting a “shrill” critique “when media concentration was quite low.”^65^

Bagdikian asserted that five top media firms controlled entertainment platforms in the U.S., enjoying “more communications power than was exercised by any despot or dictatorship in history.”^66^ Yet, these firms, whatever their market power then (Noam is skeptical that they exercised such control), constitute but a tiny fraction of the value of the leading digital firms only a few years later: FAANG (Facebook, Amazon, Apple, Google, Netflix). Of course, these firms were much smaller in 2004 (Facebook did not exist, Netflix was an upstart DVD online mail-order service, and Google was just going public in 2004). The idea that great power was exercised—that the managers of the conglomerates “constitute a new Private Ministry of Information and Culture” —would prove wishful thinking were it then held by shareholders of the Old Media. By 2019, the total market value of the five firms said to control the situation in 2004 was just eleven percent of the FAANG firms seen to surpass them.

**Table 2:** Top Five Media Firms 2004 vs. 2019: Market Caps

2004 “Cartel” at 2019 value ($Bil.)		New Digital Firms in 2019 ($Bil.)		Ratio (2004/2019)
Bertlesmann	$0.97*	Facebook	$556.38	
Time Warner	$113.09	Amazon	$862.43	
Disney	$260.61	Apple	$1,180	
Viacom	$9.45	Google	$920.53	
NewsCorp	$7.70	Netflix	$129.3	
Total	$391.82	Total	$3,648.64	0.11

*Euros translated to USD at Nov. 16, 2019 exchange rate @ $1.05477 =  €1.  Firms from Ben Badigkian, The Media Monopoly, 20th Ed. (Boston: Beacon; 2004); Jason Fernando, FAANG Stocks: Definition and Companies Involved, Investopia (June 29, 2022).

## Vertical integration

One matter of controversy is the degree to which firms specialize. A producer of automobiles may decide to produce engines, but buy the steel inputs, from other companies. These decisions about the scope of the firm’s activities are the subject of much study by economists since a pioneering paper published in 1937.^67^ General Motors once purchased car bodies from an independent supplier, Fisher Body; it then acquired Fisher Body and made these components internally. This integration has been explained by economists as an efficient coordination of risky, long-term, complementary investments.^68^ At the same time, other trends go in the reverse direction, away from vertical integration. GM now reports using scores of independent parts suppliers.^69^

Tesla, a more recent industry entrant, produces both electric vehicles and the batteries that power them. This integration is tight and ambitious, with the firm building a massive Gigafactory to dramatically increase its battery production capacity. It partners with Panasonic in this effort, an “integration by contract,” and by merger, having acquired by Maxwell Technologies.^70^ Tesla attempts to exploit complementarities that will allow it to better fund, conduct, and then utilize the innovative technologies it develops. It has widened this aggregation by purchasing Solar City, a maker of solar panels. Following that merger, Tesla boasted that it had built “the world’s only vertically integrated energy company,” and would supply power to both a consumer’s house and car. Whether this entrepreneurial effort will succeed is a wager reflected in the company’s equity share price. But the competitive rivalry over business models—with Tesla’s rivals typically buying batteries from outside suppliers—is a socially valuable discovery process.

That conclusion is rendered by observation. Vertical integration is ubiquitous, even where monopoly is not an issue and efficiency is the obvious outcome. The first radio broadcasting station went on the air in Pittsburgh, Pennsylvania, Nov. 2, 1920. Who would invest in such a technology, given that there were no receivers? And, on the other hand, what household would buy a radio when there were no stations to listen to? This chicken-or-the egg dilemma was remedied by Westinghouse, which created KDKA, and its free-to-listener audio service, in order to sell its receivers. This vertical integration was efficient—there was no radio market to monopolize—and the innovation unleashed an entirely new sector.

Research by economists has considered the possibilities, examining particular market structures which appear more or less vertically integrated. Efficiency may be the result, or it is possible that company practices—by merger, or pricing, or packaging—foreclose rivalry, lessening competition. A 2005 study concluded that vertical integration was overwhelmingly associated with lower costs and better outcomes for consumers.^71^ An article in 2007 in the *Journal of Economic Literature* surveyed published academic research, and reached the same conclusion. Wrote economists Francine Lafontaine & Margaret Slade:

As to what the data reveal in relation to public policy, . . . [w]e are . . . somewhat surprised at what the weight of the evidence is telling us. It says that, under most circumstances, profit-maximizing vertical integration decisions are efficient, not just from the firms’ but also from the consumers’ points of view. Although there are isolated studies that contradict this claim, the vast majority support it. Moreover, even in industries that are highly concentrated so that horizontal considerations assume substantial importance, the net effect of vertical integration appears to be positive in many instances. We therefore conclude that, faced with a vertical arrangement, the burden of evidence should be placed on competition authorities to demonstrate.^72^

This conforms to U.S. antitrust law. Vertical integration is not a per se violation of the competition statutes. Whereas horizontal collusion (price-fixing among rivals) is considered a “naked restraint” that restricts output without offsetting benefits, trade-offs are inherent in vertical coordination. Practices that include mergers, contracts, and other coordination between producers of complementary factors, widely produce benefits. That they may sometimes produce restrictions on rivalry, say by increasing barriers to entry or enforcing horizontal price agreements, is recognized by the law. But those instances must be distinguished from the most common case in which efficiency explains the economics using a “rule of reason,” distinct from the “per se” rule governing horizontal collusion.^73^

Moreover, it is predictively disruptive for each and every integration decision by a firm to be subject to regulatory oversight, which would act as a tax on productive activity. Hence, the “Government has the burden of proof to demonstrate that the merger is likely to lessen competition,” wrote Judge Richard J. Leon in his 2018 opinion in *U.S. v. AT&T*.^74^ Therein, the U.S. Department of Justice challenged a vertical combination, with AT&T (a major distributor of cable TV programming, through telecommunications networks and its subsidiary, DirecTV) bidding to acquire Time Warner (a major producer of cable TV programming, including CNN, HBO, TNT, TCM, TBS HLN and the Cartoon Channel). A “rule of reason” analysis in the opinion led Judge Leon to rule that the Government had advanced a plausible theory of vertical foreclosure, but that evidence was needed to support the asserted outcome:

… evidence indicating defendants’ recognition that it could be possible to act in accordance with the Government’s theories of harm is a far cry from evidence that the merged company is likely to do so (much less succeed in generating anticompetitive harm as a result).^75^

The court ruled that such evidence was not offered, and transaction was permitted. The legal outcome was not difficult to forecast. Such claims of consumer harm as were made in the vertical merger case are difficult to establish, and legal scholars have noted that similar cases against vertical mergers are rare.^76^ The Government’s argument was that combining Time Warner’s network program ownership with AT&T’s retail video subscriber business would allow the merged firm to raise wholesale prices (license fees) on the cable networks sold to other distributors. Should those cable or satellite operators (say Comcast or DISH) resist, AT&T could terminate their program access and reap some benefit in higher DirecTV subscription take-up (as AT&T’s subsidiary would have network shows not available on rival systems). Yet Time Warner had previously been integrated with a major cable TV service provider—Time Warner Cable—and had voluntarily chosen to spin the subsidiary off into a separate, stand-alone operator in 2009. This divestiture sacrificed whatever such strategic ploys were available from integration, suggesting that the benefits of “foreclosure” were illusory.^77^

## Digital platforms

Much interest revolves around the competitive issues now concerning digital platforms. These institutions bring to the fore the conflicts between the two primary ways we tend to think about competitive enterprise:

1. as an equilibrium where the state of competition reflects market concentration;
2. as a process where rivalry to innovate produces corporate winners and losers.^78^

It may seem straightforward that firms with high market shares (as in the competitive model in [1]) tend to under-perform from a Consumer Welfare perspective; that is, with dominance, such companies are not incentivized to improve prices, customer service, or products. Yet, it is not. A major theme in economic theory for a century or more is that firms compete over time to achieve dominant market positions, and that this quest encourages (and rewards) risky investments undertaken to innovate. “Schumpeterian competition” focuses (as in [2]) on the social gains from this process to achieve market power, discovering superior business models and technologies as the path to profit.^79^

Indeed, one of the most important recent topics in business economics is the problem innovative firms confront when they improve products or market structures. An important 1986 article by David Teece attempted to “explain why innovating firms often fail to obtain significant economic returns from an innovation, while customers, imitators and other industry participants benefit.” The observed approach is “for the innovating firm to establish a prior position in these complementary assets.”^80^ Where successful, this strategy bridges the gap between risk and return, promoting the creation of socially valuable assets.

Hence, platforms are often reliant on vertical integration. Firms tend to diversify at different, key points in an ecosystem such that the beneficial outcomes they create pay off for their shareholders as well as for others. This incentivizes innovation with a feedback loop that fuels social progress.

Nested within this general approach are a wide variety of industrial structures and business strategies. It is noteworthy that many of the complementary investments that spur platform creation are made via non-profit contributions on “open platforms.” Jonathan Barnett documents how many firms have literally given away property rights to key technologies in order to help invigorate the cooperation of other firms—“strategic forfeiture.” IBM developed and then put valuable UNIX computer code into the public domain, seeking to build complementary assets that would stimulate demand for its mainframe computers. Nokia undertook a similar approach in vesting its Symbian mobile operating system software in a non-profit foundation that was owned by multiple stakeholders (including Nokia). This was undertaken to promote Nokia’s mobile devices.^81^

The terms and conditions on which the technologies are spun off (including the nature of the “open” licenses issued for intellectual property) are determined by the divesting owner. More deeply, the actions show the far-reaching importance to the innovator in gaining broad acceptance of its platform, and how it is key for such firms to position their investments in multiple spaces in an emerging ecosystem. It is this diversification that spontaneously leads to vertical integration as part and parcel of an efficiency-creating enterprise.

## Vertical integration in the digital economy

This section, through brief description of selected episodes, illustrates how emerging digital platforms have benefited from vertical integration.

### Apple iTunes

In the early 2000s, music was being distributed over the Internet, but there were fundamental problems respecting standards, safety, and intellectual property. In explaining the issues, Jack Goldsmith and Tim Wu focused on Kazaa, a software application that allowed peer-to-peer file transfers. It was designed to be radically decentralized, in part to defray liability for copyright infringement. But “Kazaa had endless problems policing bad users who fake files, porn ads, and other abusive content on the network.”^82^ Spyware and viruses were endemic, while copyright lawsuits from the Recording Industry Association of America raised piracy issues.

The situation was chaotic, what some might call “market failure.” But a solution was soon to emerge. “Instead of going to war with the recording industry,” wrote Goldsmith and Wu, Apple “struck a deal” with them.^83^ The computer company introduced iTunes in April 2003. While some skeptics scoffed at the idea of charging $0.99 a song (*how can you beat free*?) or the relatively small catalogue of iTunes titles at launch (200,000), the popularity of the new service was overwhelming. In the first week, one million songs were purchased.^84^ By June 2005, Apple sales were outpacing all the peer-to-peer services, and the company, in financial distress just a few years before,^85^ was on its way to becoming the highest valued firm in the world.

The iTunes venture was an integration from Apple’s computer business. And Apple’s iPod, a digital music player, was linked: iTunes was the one place customers could download content. This exclusivity was a key portion of Apple’s strategic effort, extending the company’s long-standing reliance on producing complementary products in-house. This was hugely controversial, prompting none other than Bill Gates to urge Apple to open its ecosystem, taking on computer producing partners, in a now famous 1985 letter. Gates told Apple executives that they needed to “make Macintosh a standard,” but that no company—not even IBM—could do that alone. His interest at Microsoft, not to go public until the following year, was to expand Apple’s software platform where Microsoft’s applications were prominently displayed.^86^

The iPod/iTunes bundle was hugely successful, and quickly attacked by European antitrust authorities.^87^ That was because “the subscription to iTunes forces a consumer to purchase an iPod to enjoy the downloaded music on a portable music player.”^88^ The integrated approach followed Apple’s general integration strategy, but was seen as a threat to competition. It was not. Rival platforms were available from Samsung, Sony and others, and over the long haul the tight integration has given way to alternative arrangements. But the packaging helped create a platform, and soon an entire industry was born.

### Amazon’s eCommerce platform^89^

Amazon was launched as a project to sell books online. When that went well, it integrated into countless other product markets, becoming “The Everything Store.”^90^ It has emerged as the world’s most valuable brand,^91^ and it finished 2019 as one of the top three most valuable companies globally.

Lina Khan argues that Amazon’s platform is enmeshed in “anticompetitive conflicts of interest.”^92^ In hosting third party vendors to sell products on Amazon, for instance, the host monitors product sales and observes prices charged—“the company has used ‘insights gleaned from its vast Web store to build a private-label juggernaut.’”^93^ Overall, “Amazon seeks to cut out the independent seller.”

**Fig. 1.**
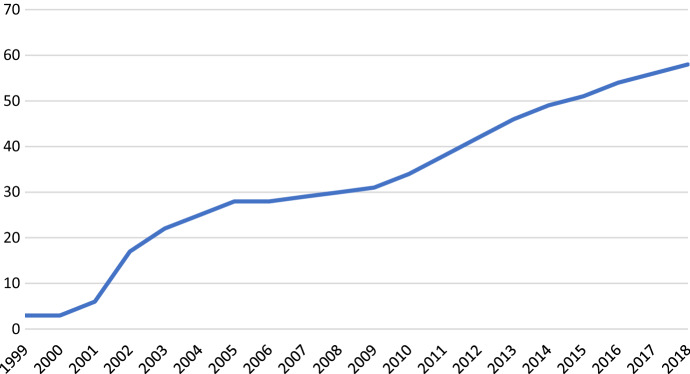
Percent of Amazon Gross Platform Sales by Non-Amazon Sellers. Source: 3rd-party sellers are thriving on Amazon, Business Insider (May 13, 2019)

In 1997, the year Amazon issued its Initial Public Offering, 97% of the product sales on the Amazon website were supplied by Amazon itself. In 2018, just 42%. See Fig. 1. Rather than stealing lucrative markets from retail vendors, Amazon has grown large by building a platform hosting independent vendors who, in turn, pay for Fulfilllment-by-Amazon (services provided to vendors by Amazon) because of the efficient sales platform offered. Amazon profits from this trade volume, earning about one-quarter of gross third-party sales via commissions and services (including shipping), about $40 billion in revenues in 2019.^94^

Were Amazon appropriating these sellers, it would be curious why Amazon was developing such a popular sales service for third party vendors. A rival platform has existed in eBay, a firm that in 2005 was three times the market capitalization of Amazon.^95^ eBay is a “pure” reseller, auctioning only merchandise sold by independent firms, and hence avoids the mixed incentive conflicts asserted to undercut Amazon’s platform. Yet, Amazon accounts for nearly twice the gross merchandise volume as eBay, counting only non-Amazon vendors, today.^96^

Vertical integration led Amazon to innovate in markets beyond eCommerce, most notably in the launch of Amazon Web Services (AWS), also known as “the cloud.” This extension of the platform began in 2006, offering firms and individuals access to high-capacity data storage, retrieval, and processing services. It is has proven highly profitable, and AWS is now seen by financial analysts as comprising roughly one-half of total Amazon capital value.^97^ As Amazon CEO Bezos thought his firm enjoyed “a natural advantage in its cost structure and ability to survive in the thin atmosphere of low-margin businesses.”^98^ In 2019, Amazon accounted for 47% of global cloud revenues, with Microsoft Azure at 22%, Alibaba 8% and Google 7%.^99^

The cloud serves to reduce important barriers to entry in the economy generally, a strategic goal motivating the integration. As described by Bezos:

The best analogy that I know is the electric grid. You go back in time a hundred years, if you wanted to have electricity, you had to build your own little electric power plant, and a lot of factories did this. As soon as the electric power grid came online, they dumped their electric power generator, and they started buying power off the grid. It just makes more sense. And that’s what is starting to happen with infrastructure computing.^100^

### AOL

One of the key contributions enabling the emergence of the mass-market Internet involves the promotion of dial-up Internet access by America Online in the 1990s. AOL promoted easy-to-use sign-up disks, making access attractive to those without a technical bent. It also created proprietary program services, putting subscribers in a “walled garden” available only to AOL members. This vertically integrated, exclusive environment changed over time, as AOL’s Internet service scaled back to supplying simply network access, responding to the growth of non-AOL content and the demands of its customers to go straight to the public Internet. But the success of the company, which became the largest Internet Service Provider by the end of the millennium, was instrumental to the growth of the overall ecosystem.^101^

Another important vertical integration offered by AOL was Instant Messaging. The service became extremely popular, and by the time of the 2001 merger between AOL and Time Warner, had attracted a 140 million users. The Government, in initially opposing the merger (the Federal Trade Commission filed its opposition, and then permitted the transaction based on certain conditions), mandated that AOL’s IM offer interconnection to rival messaging services. These terms proved irrelevant. AOL’s asserted market power, by itself or via a vertical relationship with the country’s largest ISP, could not sustain the service against new rivals that were *not* interconnected. Today, computer and mobile device users have a wide variety of messaging services to use; some are integrated with other services (texting, Facebook messaging, Google messaging, etc.) and yet they are not linked (inter-connected) to their rivals. Alas, AOL IM pioneered an important idea, and its vertical strategy was benign.

## Broadband and mobile networks

There is long-standing controversy surrounding the competitive position of the U.S. in global broadband services. The policy discussion often concludes that America, from where the Internet was launched, has lost its mojo.^102^ In 2004, President George W. Bush, opined that the U.S. was then ranked tenth among all countries in terms of broadband subscriptions per capita, and curiously summarized the problem: “Tenth is ten spots too low.”^103^ A decade later, law professor Susan Crawford calibrated things differently, but evinced the same basic sentiment: “Americans aren't quite aware of it because we don't look beyond our borders, but we're falling way behind in the pack of developed nations when it comes to high-speed Internet access, capacity and prices.”^104^

Tyler Cowen, in his 2019 book, Big Business: A Love Letter to an American Anti-Hero, provides an energetic defense of capitalism, American Style. Yet, he concedes as how three sectors of the U.S. economy may be overly concentrated and insufficiently competitive: hospitals, cable television, and mobile services.^105^ I leave the issue of hospitals, which Cowen notes operate under extensive regulatory constraints, for another discussion. Here I explore the competitive issues in Broadband and Mobile markets, with Cable TV to be a subset of the former and increasingly, the latter. Even to champions of Big Business, these may appear problematic under our current policy regime.

Both markets are concentrated, relative to most others, but the basic question remains: is competition forcing efficiencies, including economies of scale, or being thwarted? Are there better policies we should be adopting, including those demonstrated as superior in other markets? More rivalry, including the introduction of new networks, is better—all else equal. But suppose regulators were to, in a quest for greater rivalry, break up broadband Internet Service Providers into smaller overlapping competitors? Better yet, if spectrum allocators at the Federal Communications Commission could simply set sharp(er) limits on how much wireless bandwidth a mobile carrier could control, forcing there to be, say, ten or twenty mobile carriers in each market. Would that effectively promote competitive market forces?

**Fig. 2:**
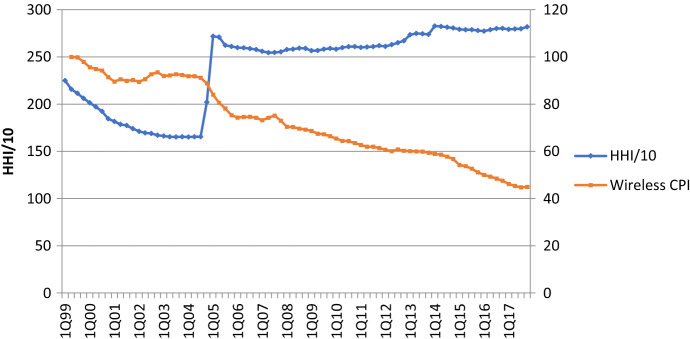
US Mobile Market HHH/10 & Wireless CPI

In fact, it would clearly subvert them, by forcing costlier structures on the market, punishing consumers and stifling innovation. The compatibility of concentration growth and declining prices is not an anomaly, but has been observed in the U.S. market, where mobile networks have expanded services, upgraded technologies, and consolidated, even as prices were falling dramatically. In Fig. 2, the trend in the national concentration ratio (divided by ten to allow a better graphical comparison) is shown with the trend in retail prices for mobile services, Wireless CPI. The HHI data are from Bank of America/Merrill Lynch, while the Consumer Price Index wireless component is collected by the Bureau of Labor Statistics. The time period is 1999 through 2017.

The reduction in prices throughout the period is pronounced, even as network consolidation is clearly occurring. Much of this consolidation linked firms operating in distinct geographic markets, an illustration of how aggregate concentration data can misleadingly suggest concentration within markets. Yet the creation of large national networks was itself a contributor to growth, and a significant part of the consolidation was between direct competitors. And, indeed, during 2004–2005, two major mergers—Sprint-Nextel and Cingular-AT&T Wireless—reduced the number of major national carriers from six to four. HHI jumps accordingly, and is here capturing a reduction in the number of direct rivals.

The more concentrated market did not result in less competition but more. Not only do prices fall fairly rapidly in the wake of the transaction, the mergers were part and parcel of a move to adopt 3G (third generation) technology. This introduction of wireless broadband was facilitated by the more economical use of spectrum in four, as opposed to six, carriers. Some policy analysts (including the author of this report) argued that regulators should open access to much more generous bandwidth, overcoming a decade-long drought in newly auctioned airwave rights. That would have also have helped to push 3G into the market, promoting efficiencies. But the outcome—under such restrictions—is fairly clear: consolidation was consistent with increased investment in new wireless technologies, large improvements in network quality, substantial gains in wireless usage (including for texting and data in addition to voice minutes), and falling real prices for consumers.

This result is not surprising: no country forces “atomistic” competition on broadband or wireless markets. European countries, for instance, generally feature two, three or four national networks, not ten or twenty. Fixed broadband tends to be more concentrated. While artificial entry barriers should be eliminated, forcing uneconomic deconcentration would sacrifice low cost supply and, barring state-paid subsidies, low cost service.

### Broadband

Both wired (fixed) and wireless networks serve high-speed data to end users, but “broadband” is often taken to refer to the former. I will follow that custom here and discuss mobile competition just below.

The U.S. market produced mass market access to the Internet when narrowband Internet Service Providers such as Prodigy, CompuServe and America Online (AOL) entered the market. They were not common carriers, were not licensed, and were not mandated to provide service as regulated telephone companies (with universal service obligations, tariffed prices, and tax levies in the form of access fees). These services took off with privatization of Internet transport facilities and the commercialization that followed in the 1990s. The enthusiastic response by subscribers, as well as by content and application developers in creating an ecosystem for online services, led unregulated cable TV systems to launch a competing product, broadband access, in the latter part of the 1990s. The disruptive event was assisted by the 1996 Telecommunications Act, which overturned state monopoly telecommunications franchises. This, in turn, spurred regulated telephone carriers—long technically able to provide faster Internet access, but slow to roll-out—into action. A race to wire the market ensued.^106^

**Fig. 3:**
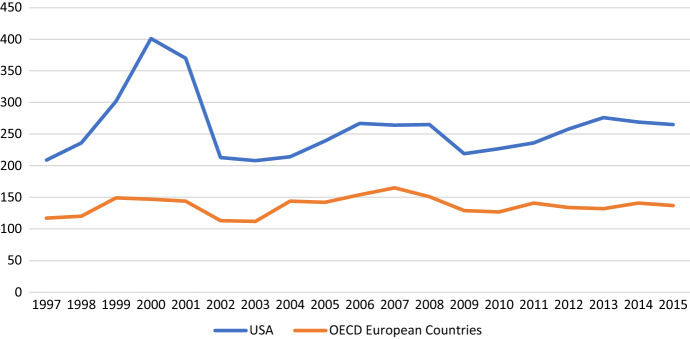
Per Capita Telecoms Infrastructure Investment, USA v. Europe (1997–2015). Source: OECD and USTelecoms. European group excludes Latvia, Slovenia, the Slovak Republic and Sweden due to missing data

Alternative pathways to competition exist. In the U.S., policies have tended to encourage market rivalry between multiple carriers—cable v. telephone, and now fixed v. wireless.^107^ Alternatively, European market economies have been more dependent on monopoly carriers and have sought to promote competition via mandated network sharing rules. These appear to have discouraged competition. In a 2012 study Michal Grajek and Lars-Hendrik Röller found that higher levels of regulatory control undermined investment incentives, reducing information infrastructure across Europe by 23%.^108^ These rules have been attempted in the U.S. and largely resisted or repealed, forming the crux of the “under-regulated” thesis. But (and) U.S. network investments are higher than in Europe, even accounting for higher U.S. GDP. Over 1997–2015, ninety percent more was expended on capital for telecommunications infrastructure in the U.S. See Fig. 3.

**Fig. 4:**
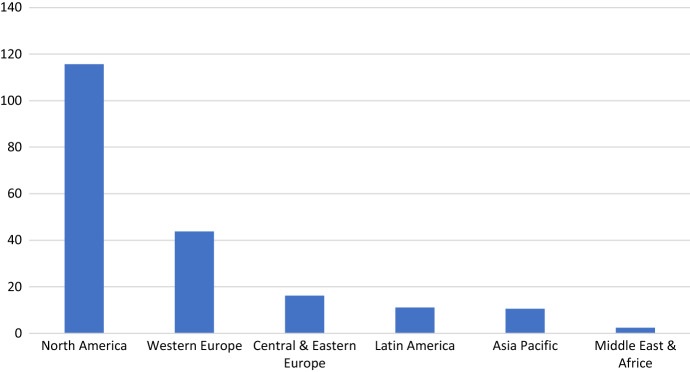
Global Data Usage Per Capital per Month (2017). Source: Cisco and USTelecoms

This has been a salubrious outcome for U.S. networks, U.S. innovation, and U.S. Internet households. American Internet users consume considerably more data than do Europeans on a per capita basis. These Cisco data in Fig. 4 lump the U.S. and Canada together in the “North America” category (Mexico is in “Latin America”), but convey the general point. It has been noted that, during the current pandemic causing surges in data usage, U.S. networks have survived the “stress test” relatively well, while European networks have undertaken to reduce traffic flows by asking Netflix and YouTube to reduce bandwidth use by lowering the definition of videos.^109^

A key driver in this embrace and development of the broadband Internet has been the transformation of electronic media markets. Broadcast television dominated U.S. video distribution through the 1970s, as federal policies protected licensed broadcasters from competition among themselves (establishing the long-running network triopoly of NBC, CBS and ABC) and from new media such as satellite^110^ and cable television. The latter was blocked by FCC rules enacted in 1962, 1965 and 1966, on the reasoning that the “public interest” would be served by stopping an ancillary service that would never provide market-wide service but might detract from the financial viability of TV stations.^111^ The result was that Americans were given little to choose from on the television dial.

That changed dramatically following the deregulation of cable TV in the late 1970s, and the wiring of America for competitive alternatives by the mid-1980s. Dozens, then hundreds, of new program networks materialized. Policies were again imperfect—cable TV franchises imposed by municipalities thwarted progress and resulted in considerable rent-seeking waste^112^—but the emergence of inter-modal rivalry pitting broadcast against cable left the old (artificial) scarcity behind. The trend was bolstered when satellite TV was finally able to gain market access in the two nationwide launches of DirecTV (1994) and DISH (1996).

When broadband rivalry erupted between the cable systems and the legacy phone carriers in the early 2000s, yet another new world would soon open for video content: over-the-top. It is important to see this in the long-run context of open market policies. Rivalry between program distributors (cable and phone carriers are identified as Multichannel Video Program Distributors, MVPDs, when they deliver video to subscribers) led these networks to upgrade to expand capacity.^113^

**Fig. 5:**
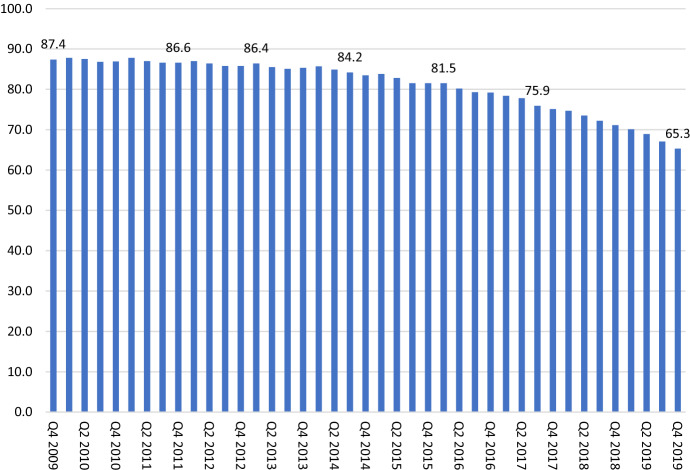
Traditional Pay TV Penetration (% Occupied Homes). *Source*: Moffatt Nathanson, *Cord Cutting: The Great Unwind* *Call Deck* (Feb. 26, 2020), 5

That service is now in the process of consuming its benefactor. The hosts of “over-the-top” media are the underlying broadband networks, cable and telco operators which integrated into the Triple Play: voice, phone and video. But the capacity brought forth to serve that purpose has unleashed rivalry in additional dimensions. Innovation is pushing customers to next generation video. Traditional “Pay TV” services are rapidly declining. See Fig. 5. Over the 2009–2019 decade, the percent of U.S. households not subscribing to basic cable TV service increased from 12.6 to 34.7%.

Of course, this “gap” is largely pushed open by Netflix—now subscribed to over half of U.S. television households.^114^ This remarkable migration is testimony to the openness of markets to innovative business models, as Netflix was an upstart that entered the DVD rental business, pursuing online sales with delivery through the mail, against an array of vastly larger, well established enterprises including Blockbuster, Amazon, Apple, Best Buy, Circuit City, and Target, as well as potential competitors Time Warner and Disney, and cable operators such as Comcast (integrated into services like pay-per-view).^115^ Indeed, Blockbuster, with its 9000 retail outlets and a ubiquitous presence in video rentals (judged to be potentially monopolistic in 2005 when the FTC nixed an attempted merger with Hollywood Video) engaged Netflix in a brutal price war launched in late 2004. The then tiny Netflix survived; the giant Blockbuster went bankrupt in 2010. For all the power of incumbency, none of the incumbents in this market seized the opportunities that an entrant captured or could block Netflix from profiting. And now the incumbents are racing to catch up.^116^

Now the challenge to Netflix is widespread, with offerings including Sling, YouTube TV, PlayStation Vue, Apple TV, Amazon Fire and Hulu seeking to provide vMVPDs (mimicking program channel line-ups of traditional TV, but delivered virtually via broadband). And a wide array of competing streaming video services abound, including Amazon Prime, Peacock, and Quibi, while Disney and HBO (Time Warner) are “new” entrants. The dynamism is a product to the underlying regime. The gains to consumers duly noted. As should be the benefits also distributed to the arms manufacturers in this war: Hollywood producers.^117^ It is a new Golden Age for video content, fueled by creative destruction in delivery markets, expanding capacity to serve demands. And the inputs are being frantically supplied, as reported by the *N.Y. Times*:

All of our screens are now TVs, and there is more TV to watch on them than ever. More dramas, more comedies, more thrillers, more fantasy-adventure series, more dating shows, more game shows, more cooking shows, more travel shows, more talk shows, more raunchy comedies, more experimental comedies, more family comedies, more comedy specials, more children’s cartoons, more adult cartoons, more limited series, more documentary series, more prestige dramas, more young-adult dramas, more prestige young-adult dramas—more, more, more.^118^

### Mobile

Mara Faccio & Luigi Zingales write that “If there is a sector where the government can affect the degree of competition, the mobile communication industry is one.”^119^ The point is well taken, given that radio spectrum allocations are products of government policy, and these processes heavily influence how wireless markets develop. The authors proceed to argue that U.S. competition policy has been too lax, allowing market concentration to go too far, and that competition policy in Europe has been tougher and more pro-consumer.

NYU economist Thomas Philippon’s The Great Reversal: How America Gave Up on Free Markets (2019) continues this line of thought. The book was motivated by the author's observation that European economies have advanced way past the U.S. in—as his first example— “home interest access.” As evidence, he points to mobile wireless, citing a survey indicating that U.S. households paid about $66 a month for residential broadband while just $36 in Germany, he asks, “How did the U.S…. become such a laggard…?”.^120^

In fact, the U.S. is leading the world in Internet usage and our networks are pouring out data. There are many ways we might do better, and sudden demand surges will require new remedies (as when Costco runs out of Clorox wipes or the Zoom platform has to update security). But America’s broadband networks are performing as well as the EU’s and, in quantity measures, far better.

This comes, in part, from higher investment levels and a greater competitive push to upgrade to the latest technologies. In 2018, the news was reported this way: “Europe Will Remain a 5G Laggard, Says Ericsson Report.”

Europe is set to be one of the world's 5G laggard regions, with the technology accounting for just 30% of mobile subscriptions by the end of 2024, compared with 55% in North America and 43% in North East Asia. This is one of the predictions made in Ericsson’s latest Mobility Report,… [T]he Mobility Report measures the growth of data traffic in Q3 2018 -- up 79% year-on-year overall, with North America still registering the highest data traffic per smartphone, a figure that is set to reach 8.6 gigabytes per month by the end of this year.^121^

**Fig. 6:**
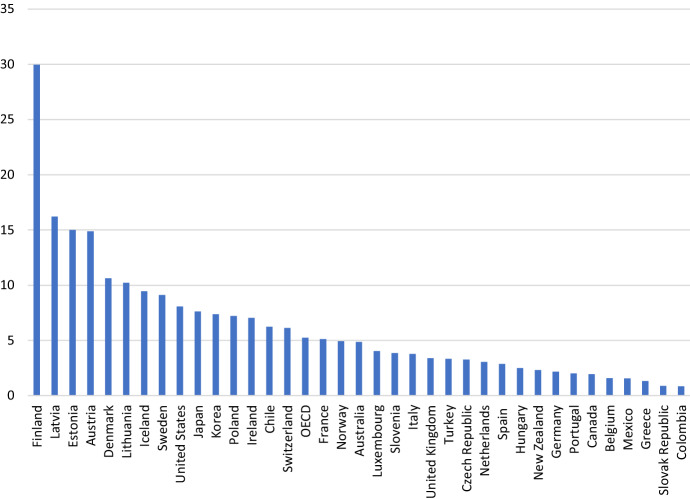
Per Capita Mobile Data Consumption (GB/mo.). Source: OECD Dec. 2018 (Mobile Usage), June 2019 (Mobile Penetration)

In using the latest OECD data for mobile penetration (what percentage of the population subscribes to wireless service) and usage (how much data each subscription consumes) we can plot average monthly mobile data usage per capita. See Fig. 6. This takes account of affordability (higher prices discourage usage), and quality of the network (better technologies and customer service encourage usage). The U.S. exhibits relatively high mobile usage, at over 8 GB per capita per month, compared to, e.g., Germany’s 2.2 GB. There are several small European countries that feature greater levels than the U.S., but no large market. And the U.S. slightly exceeds Japan and South Korea, as well.

One important observation is that Finland is a star performer in wireless, and we can learn from their policy experience. The country, while operating with three national mobile networks (the U.S. has operated with four since the consolidation from six in the mid-2000s) has been extremely aggressive in allocating more radio spectrum.^122^ This liberalization is likely driven by the country’s strategic push to advance wireless technologies, given comparative advantage in this sector (home to Nokia), but the message in the outcome is available for other countries including the U.S. To stimulate efficiencies leading to great outputs and a range of productivity gains, remove restrictions and auction more bandwidth rights with technological neutrality.

**Fig. 7:**
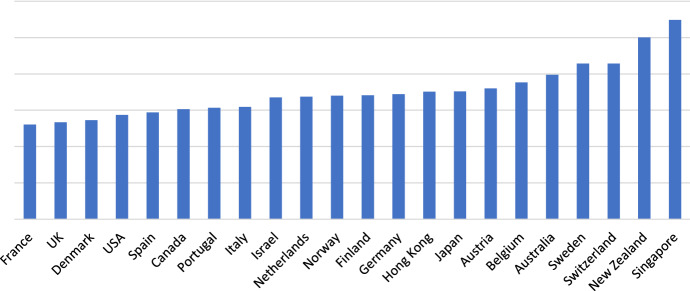
Mobile Sector HHIs in Developed Markets, 20193Q (Bank America/Merrill Lynch data)

In addition to boasting advanced mobile networks serving large volumes of data, U.S. markets are relatively unconcentrated. This goes against the arguments made by Faccio & Zingales, for example, who argue that U.S. performance suffers from a lack of “antitrust activism.”^123^ In Fig. 7, HHI metrics are shown from Bank of America/Merrill Lynch (2019 3^rd^ quarter) for the top global economies. The U.S. ranks fourth from the bottom (least concentrated) at 2871, while Germany, for instance, is higher at 3440. It is difficult to see where competition policy has been under-supplied in the U.S., particularly when time trends are shown that establish that the U.S. has consistently been among the least concentrated markets over the past two decades.

In sum, U.S. markets for broadband are open to policy reforms that improve efficiency and competitiveness, but replacing U.S. law with the regimes that are visibly less successful in promoting innovation and competitive rivalry does not appear a winning strategy. In fact, the U.S. is overwhelmingly productive in the ecosystem that has built up around broadband infrastructure in the Internet. Among Mary Meeker’s Top 30 Internet-related firms globally, 18 are U.S. based, including the top five (Microsoft, Amazon, Apple, Alphabet, Facebook), and only one is from Europe (Spotify, at Number 30, is Swedish).^124^ Innovation in the wireless space is perhaps even more dramatic as two leading U.S. tech firms have created competing ecosystems—Apple with the iPhone, iTunes and the App Store; Google with Android mobile operating system and Google Play—that dominate world markets. These innovations overlay mobile networks, and uprooted previous ways of organizing telephone communications. Nokia, the world’s leading smartphone maker, and RIM Blackberry, an entrepreneurial Canadian firm that pioneered the overlay network concept with its addictive handsets in the 2000s—paid the competitive price. But consumers won. An estimated 3.5 people own smartphones, 45% of the world’s population.^125^

## Conclusion

Business model competition shapes markets en route to the discovery of varied and surprising forms of competitive superiority. The experimentation is valuable. Where anti-competitive outcomes result, it will—as per wide observation and empirical research—be the exception rather than the rule. Where adjustments in policy may be made to improve competitive outcomes, they ought surely be implemented. But suppressing incentives for innovation by categorically racheting up antitrust enforcement risks errors that are decidedly weighted against efficiency and consumer welfare. Perhaps Richard Langlois states this more eloquently: “Proponents of anti-tech antitrust [must] explain why consumers [are] being harmed by an incomprehensibly magical information source [with] swift access to virtually all the products of humanity at the touch of a finger [at] quality-adjusted prices that continue to plunge through the floor.”^126^

## Footnotes

1 Erik Brynjolfsson & Joo Hee Oh, *The Attention Economy: Measuring the Value of Free Digital Services on the Internet*, 33rd International Conference on Information Systems (Orlando, 2012); Jay R. Corrigan, Saleem Alhabash, Matthew Rousu and Sean B. Cash, *How much is social media worth? Estimating the value of Facebook by paying users to stop using it*, PLoS ONE 13(12) (Dec. 19, 2018).

2 Austan D. Goolsbee and Peter J. Klenow, *Internet Rising, Prices Falling: Measuring Inflation in a World of E-Commerce*, 108 AEA Papers and Proceedings (2018), 488–92; Peter Cohan, *How Amazon Overpowers The Fed*, Forbes (Aug 25, 2017).

3 Social media have, in particular, extended rewards but posed new risks that individuals, families and institutions must deal with. See, e.g., Hunt Allcott, Luca Braghieri, Sarah Eichmeyer, & Matthew Gentzkow, *The Welfare Effects of Social Media*, Working Paper (Nov. 8, 2019).

4 Robert Crandall & Thomas W. Hazlett, *Telecommunications Policy Reform in the United States and Canada*, in Martin Cave and Robert Crandall, eds., Telecommunications Liberalization on Two Sides of the Atlantic (Washington: AEI-Brookings Joint Center for Regulatory Studies; 2001), 8–38.

5 Thomas W. Hazlett, *A Lesson for Today’s Trustbusters*, Wall Street Journal (Jan. 9, 2020).

6 “[T]the mobile marketplace two years ago was on the doorstep of duopoly. But our rejection along with the Justice Department of the proposed AT&T-T-Mobile deal, and other pro-competition actions we’ve taken, have led to an improving competition picture in the United States.” Julius Genachowski, *Prepared Remarks of FCC Chairman Julius Genachowski*, presented at The Global Internet at a Crossroads (2012).

7 *United States v. AT&T Inc*., 310 F. Supp. 3d 161, 254 (D.D.C. 2018).

8 United States v. AT&T Inc., 916 F.3d 1029 (D.C. Cir. 2019).

9 Joshua D. Wright & Jan M. Rybnicek, United States v AT&T/Time Warner: a triumph of economic analysis, 6 Journal of Antitrust Enforcement (2018), 469–477. See also, Thomas W. Hazlett, *Why the government will lose to AT&T*, Reuters (March 23, 2018).

10 Shane Greenstein, How the Internet Became Commercial: Innovation, Privatization, and the Birth of a New Network (Princeton, 2016).

11 Jason Oxman, *The FCC and the Unregulation of the Internet*, Office of Plans & Policies Working Paper No. 31, Federal Communications Commission (July 1999).

12 Expanding and liberalizing spectrum allocations would broadly improve wireless (including broadband) competition. Relaxing local barriers to entry, including through franchise requirements and permitting of network access points, would similarly expand competitive forces. See Thomas Hazlett, The Political Spectrum: The Tumultuous Liberation of Wireless Technologies, from Herbert Hoover to the Smartphone (Yale, 2017).

13 As suggested in Lina M. Khan, *The Amazon Antitrust Paradox*, 126 Yale Law Journal 710 (2017), and Tim Wu, The Curse of Bigness: Antitrust in the New Gilded Age (Columbia Global Reports, 2018).

14 Recognizing the challenges of heavily regulated systems, including those relying on traditional public utility rules, some economists suggest a “lightly regulated” system to complement antitrust law. See William P. Rogerson & Howard Shelanski, *Antitrust Enforcement, Regulation, and Digital Platforms*, 168 U. Pa. L. Rev. 1911 (2020).

15 Jason Furman, *Walmart: A Progressive Success* Story (Nov. 28, 2005).

16 Sebastian Mallaby *Progressive Wal-Mart. Really.* Washington Post (Nov. 28, 2005).

17 Deirdre Nansen McCloskey, *The Great Enrichment: A Humanistic and Social Scientific Account*, Scandinavian Economic History Review (Spring 2016).

18 The assessment is a correct one, although the magnitude of the damage may be sometimes overstated, as per Kenneth G. Elzinga & Thomas Hogarty, *Utah Pie and the Consequences of Robinson-Patman*, 21 Journal of Law & Economics 427 (Oct. 1978).

19 *United States v. Griffith*, 34 U.S. 100 (1948), 108.

20 Frank H. Easterbrook, *The Limits of Antitrust*, 63 TEXas Law Review 1 (1984).

21 Joshua D. Wright, Elyse Dorsey, Jonathan Klick and Jan Rybnicek, *Requiem for a Paradox: The Dubious Rise and Inevitable Fall of Hipster Antitrust*, 51 Ariz. St. U. L. Rev. 293 (2019).

22 Quoted in Thomas M. Jorde and David J. Teece, *Innovation and Cooperation: Implications for Competition and Antitrust*, 4 Journal of Economic Perspectives (Summer 1990), 75.

23 Ibid., 78.

24 Assistant Attorney General Makan Delrahim Delivers Keynote Address at American Bar Association’s Antitrust Fall Forum, United States Department of Justice (Nov. 16, 2017).

25 Robert W. Crandall & Clifford Winston, *Does Antitrust Policy Improve Consumer Welfare? Assessing the Evidence*, 17 Journal of Economic Perspectives 3 (Fall 2003).

26 Ibid., 8.

27 Ibid., 12.

28 How happy an “exception” is still a matter in dispute. As Robert Crandall & I have written, Canada managed to introduce competition in long distance services more rapidly than did the U.S. and yet did not order a divestiture of Bell Canada, a privately-owned monopoly network similar in structure and dominance to AT&T. Robert Crandall and Thomas W. Hazlett, Crandall, *Telecommunications Policy Reform in the United States and Canada*, in Martin Cave and Robert W. Crandall, eds., Telecommunications Liberalization on Two Sides of the Atlantic. (Washington D.C.: AEI-Brookings Joint Center for Regulatory Studies), 8 –38.

29 Crandall & Winston (2003), 13.

30 The classic discussion is in Clayton Christensen, The Innovator’s Dilemma: When New Technologies Cause Great Firms to Fail (Harvard Business Review Press; 1997).

31 McCloskey (2016).

32 Thomas McCraw, Prophets of Regulation (Harvard, 1984). The volume features an illuminating chapter on the regulatory arguments advanced by Louis Brandeis during his career as a lawyer and legal champion. Brandeis was instrumental in designing the Federal Trade Commission and was later appointed to the U.S. Supreme Court.

33 Ibid., 99.

34 This is sometimes claimed to be a “Chicago School” approach specific to the work of Robert Bork (and his influential, The Antitrust Paradox [Basic Books; 1978]), but Williamson was not a Chicagoan. Moreover, the Harvard Law School approach to antitrust proceeded on a parallel track, as explained in William E. Kovacic, *The Chicago Obsession in the Interpretation of US Antitrust History*, 87 University of Chicago Law Review (2020), 459–494.

35 Harold Demsetz, *Industry Structure, Market Rivalry, and Public Policy*, 16 Journal of Law & Economics (April 1973), 1–9.

36 Stratechery is a subscription newsletter popular with investors in digital markets. Thomson has been dubbed a “tech guru” by the *Financial Times*, while the *N.Y. Times* references him as “one of the most interesting sources of analysis on any subject.” *Alphaville*, Financial Times (Sept. 8, 2018).

37 *Neither and New Follow-Up, Adam Neumann Forced Out, Peloton IPOs*, Stratechery (Sept. 26, 2019). The Thompson comments quoted in this section are from this article.

38 This disclaimer is, I trust, unnecessary: This paper does not take a position on the relative prospects for any equity issue. The Thompson analysis illustrates how sophisticated observers view dynamic rivalry. And, given the competitiveness of financial markets, along with the complexity of real-world factors involved, his particular expectations may prove incorrect in their long-run application to the performance of Uber, Lyft, eBay, Amazon or any other stock.

39 This discussion tracks that found in Thomas Hazlett, “The ‘Nirvana Fallacy’ in Hipster Antitrust,” 28 Geo. Mason L. Rev. 1253 (Summer 2021).

40 Harold Demsetz, *Barriers to Entry*, 72 American Economic Review 47 (March 1982), 56.

41 Demsetz (1973), 4.

42 Harold Demsetz, *How Many Cheers for Antitrust’s 100 Yeqrs?* 30 Economic Inquiry 207 (April 1992), 207.

43 Brad Stone, The Everything Store: Jeff Bezos and the Age of Amazon (Little Brown, 2013), 56.

44 Ibid., 57.

45 Barnes & Noble, after closing most of its stores, was sold for $638 million to a hedge fund in 2019. Jordan Crucchiola, *Barnes & Noble’s Wild Ride: A Timeline*, Vulture (June 10, 2019).

46 Stone (2013), 111. Lina Khan identifies a much larger investment, in Amazon Prime, as presumptively predatory. “As with its other ventures, Amazon lost money on Prime to gain buy-in. In 2011 it was estimated that each Prime subscriber cost Amazon at least $90 a year—$55 in shipping, $35 in digital video—and that the company therefore took an $11 loss annually for each customer. One Amazon expert tallies that Amazon has been losing $1 billion to $2 billion a year on Prime member- ships.” Khan (2017), 750 (footnotes omitted). However, Prime soon became a “huge success,” generating large profits, after initially being very controversial within the company—precisely because it was difficult to create and expensive to build (Stone 2013, 188). Its success did not depend on predation, driving rival eCommerce platforms from the market, but by attracting customers—achieving scale economies rewarding the upfront investments communicating reliable customer service, low prices, and convenience.

47 Stone 2013, 55.

48 Henry Simons, A Positive Program for Laissez Faire (Chicago, 1934); *The Requisites of Free Competition*, 26 American Economic Review 68 (1936): 68–76; Henry Simons, *For a Free-Market Liberalism*, 8 University of Chicago Law Review 202 91941); George Stigler, *Extent and Bases of Monopoly*, 32 American Economic Review (Supp.) 1 (1942); George Stigler, The Case Against Big Business, 45 Fortune Magazine 123 (1952).

49 Demsetz (1973), 3.

50 Michael Smirlock, Thomas Gilligan & William Marshall, *Tobin's q and the Structure-Performance Relationship*, 74 American Economic Review 1051(Dec. 1984).

51 Sam Peltzman, *Productivity and Prices in Manufacturing During an Era of Rising Concentration*, University of Chicago & NBER (April 25, 2018).

52 Under the S-C-P approach, economists “accept the data of the momentary situation as if there were no past or future to it and think they have understood what there is to understand if they interpret the behavior of those firms by means of the principle of maximizing profits with reference to those data.” Demsetz (1973), 84.

53 Timothy J. Muris & Jonathan E. Nuechterlein, *Antitrust in the Internet Era: The Legacy of United States v.A&P,* 54 Review of Industrial Organization 651 (2019), 677.

54 Dennis W. Carlton & Jeffrey M. Perloff, Modern Industrial Organization, 4^th^ Edition (Prentice Hall, 2004), 268.

55 The standard metric, the Herfindahl–Hirschman Index, or HHI, is calculated as the sum of the squared market shares (after multiplying percentages by 100 before squaring). So, if ten firms each have equal scale, serving ten percent of the market, the HHI = (10*10) * 10 = 1,000. The minimum value is zero, the maximum is 10,000 (100*100). In the case of 734 local markets, with 1468 suppliers of equal size, each firm would have a trivial share of national revenues (less than one), and the sum of the squared shares would itself be trivial.

56 Again, it may bear noting that defendants in antitrust cases would be delighted for a methodology that allows for nationwide market shares, no matter the local dynamics, as this could mask actual market power. In the cellular example, suppose half of the country saw local mergers between the A and B carriers. The industry would remain populated by hundreds of firms, and the concentration level would still be judged “Highly Competitive.” But half of the country would see their markets go from duopoly to monopoly.

57 Carl Shapiro, *Antitrust in a Time of Populism,* 61 International Journal of Industrial Organization (2018), 714–48; Gregory Werden & Luke Froeb, *Don’t Panic: A Guide to Claims of Increasing Concentration*, 33 Antitrust Magazine 74 (Fall 2018). These issues are also discussed in Timothy J. Muris & Jonathan E. Nuechterlein, *Antitrust in the Internet Era: The Legacy of United States v.A&P,* 54 Review of Industrial Organization 651 (2019).

58 *The State of Competition and Dynamism: Facts about Concentration, Start-Ups, and Related Policies*, Brookings Institution Hamilton Project (June 2018). The source of the analysis here is Autor, David, David Dorn, Lawrence F. Katz, Christina Patterson, and John Van Reenen, *Concentrating on the Fall of the Labor Share,* 107 American Economic Review: Papers and Proceedings (2017), 180–85.

59 Cowen (2019), 84.

60 Ibid., 84–85.

61 Ibid., 86.

62 Brookings (2018), 10.

63 Eli M. Noam, Media Ownership and Concentration in America (Oxford, 2009).

64 Ibid., 446.

65 Ibid., 437.

66 In Noam (2009), 19.

67 R.H. Coase, *The Nature of the Firm*, 4 Economica (Nov. 1937), 386-405.

68 As Benjamin Klein summarizes: “vertical integration is more likely when transactors make relationship-specific investments.” Klein, *The Economic Lessons of Fisher Body–General Motors*, 14 Int. J. of the Economics of Business (Feb. 2007), 1–36. This basic insight was developed in Oliver E. Williamson, *The Vertical Integration of Production: Market Failure Considerations*, 61 American Economic Review (May 1971), 112–23.

69 GM gave 133 firms its Supplier of the Year award in 2018. General Motors, *GM Honors Global Suppliers for Innovation, Quality and Performance*, News Release (May 17, 2019).

70 Vitaliy Katsenelson, *This could be the next gold mine for Tesla and other electric vehicles*, MarketWatch (Oct. 5, 2019).

71 James C. Cooper et al., *Vertical Antitrust Policy as a Problem of Inference*, 23 Int’l J. of Ind. Org. (2005).

72 Francine Lafontaine & Margaret Slade, *Vertical Integration and Firm Boundaries: The Evidence*, 45 Journal of Economic Literature (2007). A recent survey paper from the Global Antitrust Institute reports updated, and similar, findings.

73 “[T]he rule of reason now governs all vertical agreements” (footnote omitted). Louis Kaplow, *The Meaning of Vertical Agreement and the Structure of Competition Law*, Antitrust Law Journal (2016), pp. 563–630.

74 *U.S. v. AT&T*, opinion of D.C. Circuit (June 12, 2018), 3.

75 Ibid. 90.

76 “The DOJ’s recent challenge of the AT&T-Time Warner acquisition was the first vertical merger challenge that went to court in forty years.” The Global Antitrust Institute (GAI) at the Antonin Scalia Law School, George Mason University, *Comment: The Federal Trade Commission’s Hearings on Competition and Consumer Protection in the 21st Century, Vertical Mergers* (Sept. 6, 2018), p. 11.

77 Thomas W. Hazlett, *Why the government will lose to AT&T*, *Reuters* (March 23, 2018).

78 See David J. Teece, *Big Tech, Dynamic Competition, and Antitrust*, Working Paper, Institute for Business Innovation, Haas School of Business, U.C. Berkeley (Nov. 21, 2019), p. 9.

79 Joseph Schumpeter’s famous paradigm of “creative destruction” was posed as an alternative to the view that “perfect competition,” with numerous small firms selling at market prices influenced by no single firm, was the best outcome for consumers or society in general. ““[I]n capitalist reality as distinguished from its textbook picture, it is not that kind of competition which counts but the competition from the new commodity, the new technology, the new source of supply, the new type of organization (the largest-scale unit of control for instance) —competition which commands a decisive cost or quality advantage and which strikes not at the margins of the profits and the outputs of the existing firms but at their foundations and their very lives. This kind of competition is as much more effective than the other as a bombardment is in comparison with forcing a door…. [it is] the powerful lever that in the long run expands output and brings down prices…It disciplines before it attacks.” Schumpeter, Capitalism, Socialism and Democracy (Harper; 1942), pp. 84–85.

80 David J. Teece, *Profiting from technological innovation: Implications for integration, collaboration, licensing and public policy*, 15 Research Policy (1986), 285–305.

81 Jonathan Barnett, *The Host’s Dilemma: Strategic Forfeiture in Platform Markets for Informational Goods*, 124 Harvard Law Review (2011), 1861–1938.

82 Jack Goldsmith & Tim Wu, Who Controls the Internet (Oxford; 2005), pp. 116–17.

83 Ibid., 119.

84 Brian X. Chen, *April 28, 2003: Apple Opens iTunes Store,* Wired (April 28, 2010).

85 Jim Carlton, Apple: The Inside Story of Intrigue, Egomania, and Business Blunders (Times Books; 1997).

86 Ibid., 40–43.

87 Thomas Hazlett, *Antitrust regulators must listen to reason on iPods*, Financial Times (July 12, 2006), 15.

88 Eddy Hsu, Antitrust Regulation Applied to Problems in Cyberspace: iTunes and iPod (2005), 117–36, 118.

89 The argument in this section borrows from Hazlett (2021), op cit.

90 Brad Stone, The Everything Store: Jeff Bezos and the Age of Amazon (Little, Brown; 2013).

91 *Amazon beats Apple and Google to become the world’s most valuable brand*, CNBC.com (June 11, 2019).

92 Khan (2017), 717.

93 Ibid., 782 (quoting an e-commerce analyst).

94 *Amazon Gross Merchandise Volume $277 Billion in 2018*, Marketplace Pulse (April 12, 2019).

95 Stone (2013), 194.

96 Natalie Gagliordi, *eBay beats Q4 expectations, GMV down 5%, eBay said gross merchandise volume was down 5% year over year to $23.3 billion*, Between the Lines (Jan. 28, 2020).

97 *How Much Is Amazon Web Services Worth On A Standalone Basis?* Forbes (Feb. 28 2019).

98 Stone (2013), 221.

99 Katy Stalcup, *AWS vs Azure vs Google Cloud Market Share 2019: What the Latest Data Shows,* Park My Cloud (April 30, 2019).

100 Stone (2013), 221.

101 *See* Kara Swisher, AOL.com aol.com: How Steve Case Beat Bill Gates, Nailed the Netheads, and Made Millions in the War for the Web (Crown Business, 1998).

102 Thomas W. Hazlett, *We’re Number 2?* Commentary (Dec. 2009).

103 Cited in Thomas W. Hazlett, *The Broadband Numbers Racket*, Financial Times (Sept. 17, 2009).

104 *Author: When It Comes To High-Speed Internet, U.S. 'Falling Way Behind'*, NPR (Feb. 6, 2014).

105 “OK, so on the negative side of monopoly I’ve already mentioned hospitals, cable TV and cell phone contracts.” He goes on to discuss health care more generally as featuring market power problems, housing price inflation, higher education and K-12 education. These latter are discussed as problems not of business competition but government policy. Lack of affordable housing, e.g., is attributed to building restrictions and NIMBYism in major cities. Tyler Cowen, Big Business: A Love Letter to An American Anti-Hero (St. Martins, 2019), 95–96.

106 On the comparison of regulatory regimes that were applied to cable and phone companies, see Thomas W. Hazlett & Anil Caliskan, *Natural Experiments in Broadband Regulation,*, 7 Review of Network Economics 460 (Dec. 2008). On market power and competitiveness, see Thomas W. Hazlett & Dennis Weisman, *Market Power in U.S. Broadband Services*, Review of Industrial Organization 151 (March 2011).

107 This approach was not so clear between the 1996 Telecommunications and 2004; a federal court ruling at that point overturned FCC network sharing rules and the regime became distinctly less regulatory. See Michael A. Heller, *The UNE Anticommons: Why the 1996 Telecom Reforms Blocked Innovation and Investment*, 22 Yale Journal on Regulation 275 (2005); Robert S. Pindyck, *Mandatory Unbundling and Irreversible Investment in Telecom Networks*, 6 Review of Network Economics (Sept. 2007).

108 Michał Grajek & Lars-Hendrik Röller, *Regulation and Investment in Network Industries: Evidence from European Telecoms*, 55 The Journal of Law and Economics 189 (Feb. 2012): 189–216.

109 “So far, our networks have performed admirably, despite the surge in demand. The U.S. has not experienced either the shut-downs or the need to reduce video quality that other countries have. After the stress test, we’ll need to determine more specifically how the network performed, and if any weak links were found.” Blair Levin & Larry Downes, *The Internet After COVID-19: Will We Mind the Gaps?* Aspen Institute (April 15, 2020).

110 The interesting chapter in which competitive satellite TV entry was blocked by the federal government during the Lyndon Johnson Administration is told in Scott Woolley, The Network: The Battle for the Airwaves and the Birth of the Communications Age (HarperCollins, 2016).

111 This protectionist policy is described in Hazlett (2017), 13–21, 99–101.

112 As well as civil liberties violations including abridgments of free speech, as several federal courts found. See *Preferred Communications v. City of Los Angeles* 476 US 488 (1986). On monopoly franchises and rent seeking, see Thomas W. Hazlett, *Private Monopoly and the Public Interest: An Economic Analysis of the Cable Television Franchise*, 134 University of Pennsylvania Law Review 1335 (July 1986).

113 Indeed, cable TV operators were motivated to invest heavily in the expansion of their distribution networks in the late 1990s in large measure to respond to the entry of satellite TV rivals DirecTV and DISH. The immediate goal was to carry more programming to rival the larger channel line-ups of satellite. They discovered economies of scope in the capital expenditures, and rolled out subscription data services as an experiment. It worked.

114 Moffatt Nathanson, *Cord Cutting: The Great Unwind*—*Call Deck* (Feb. 26, 2020), 11. Netflix is purchased in 51% of U.S. TV households, accounting for sixty million subscriptions. Ibid., 23.

115 Famously, it was so unlikely to succeed that the founder, Reed Hastings, persisted in trying to sell the company to Blockbuster for $50 million in 2000, two years after its founding. Gina Keating, Netflixed: The Epic Battle for America’s Eyeballs (Penguin, 2012), 66–67. As of April 17, 2020, Netflix has a market capitalization of $187 billion.

116 “Since its metamorphosis in 2007 from a mail-based DVD-rental library into a streaming platform, Netflix has become an entertainment hegemon, spending heavily on original shows and movies (a reported 700 of them as of last year); minting new kinds of stars (the Tasmanian meta-comedian Hannah Gadsby, the Japanese home-organizing guru Marie Kondo); and growing its subscriber numbers to 149 million worldwide. Its rise coincides with a trend of major consolidations, including AT&T’s purchase of Time Warner and Disney’s recent acquisition of Fox’s entertainment properties. Each conglomerate is readying a new streaming platform, as is the Comcast-owned NBC Universal.” Jonah Weiner, *The Great Race to Rule Streaming TV*, N.Y. Times (July 10, 2019).

117 “In their rush to match Netflix, competitors like HBO, Hulu and Amazon are ordering a slew of content —ushering out the age of “prestige TV” and ushering in an age of anything goes…. HBO’s tightly curated cluster of shows, released seasonally and in weekly batches, no longer amounted to a tenable strategy. ‘It’s not hours a week, and it’s not hours a month,’ [said an HBO executive]. ‘We need hours a day. You are competing with devices that sit in people’s hands that capture their attention every 15 min.’ Ever more hours of overall watch-time were necessary…”.

118 Id.

119 Mara Faccio & Luigi Zingales, *Political Determinants of Competition in the Telecommunications Industry*, National Bureau of Economic Research (2017), 1–2.

120 Phillippon (2019), 6.

121 Paul Rainford, *Europe Will Remain a 5G Laggard, Says Ericsson Report*, Light Reading (Nov. 27, 2018).

122 In March 2019, at an International Telecommunications Society meeting in Ottaway, Canada, Dr. Heidi Himmanen, Chief Advisor for the Finnish Transport and Communications Agency Traficom, stated that the country had allocated some 1200 MHz of low- and mid-band spectrum for mobile services. That would be about 67% above the U.S. level. See also, Dan Robitzski, *Finland is the Mobile Data Capital,* IEEE Spectrum (Jan. 22, 2018): “[B]ecause Finland is often among the first to allocate certain frequencies for mobile usage, the country has a leg up on others that haven’t adopted the new frequencies as quickly or as effectively.”

123 Faccio & Zingales (2017), 4.

124 Mary Meeker, *Internet Trends 2019*, Kleiner Perkins (June 11, 2019), 12. The ranking is by market capitalization.

125 *Smartphone users worldwide 2016-2021*, Statista (Feb. 28, 2020).

126 Richard Langlois, *Hunting the Big Five*, 23 Independent Review (W 2018/19), 411.

